# Psychological stress creates an immune suppressive environment in the lung that increases susceptibility of aged mice to *Mycobacterium tuberculosis* infection

**DOI:** 10.3389/fcimb.2022.990402

**Published:** 2022-09-16

**Authors:** William P. Lafuse, Qian Wu, Naresh Kumar, Noushin Saljoughian, Shrayes Sunkum, Omar Santiagonunez Ahumada, Joanne Turner, Murugesan V. S. Rajaram

**Affiliations:** ^1^ Department of Microbial Infection and Immunity, The Ohio State University, Columbus, OH, United States; ^2^ Host Pathogen Interactions Program, Texas Biomedical Research Institute, San Antonio, TX, United States

**Keywords:** social stress, aging, *Mycobacterium tuberculosis*, inflammaging, granuloma, IL10

## Abstract

Age is a major risk factor for chronic infections, including tuberculosis (TB). Elderly TB patients also suffer from elevated levels of psychological stress. It is not clear how psychological stress impacts immune response to *Mycobacterium tuberculosis* (*M.tb).* In this study, we used social disruption stress (SDR) to investigate effects of psychological stress in young and old mice. Unexpectedly, we found that SDR suppresses lung inflammation in old mice as evidenced by lower pro-inflammatory cytokine levels in bronchial lavage fluid and decreased cytokine mRNA expression by alveolar macrophages. To investigate effects of stress on *M.tb* infection, mice were subjected to SDR and then infected with *M.tb*. As previously reported, old mice were better at controlling infection at 30 days than young mice. This control was transient as CFUs at 60 days were higher in old control mice compared to young mice. Consistently, SDR significantly increased *M.tb* growth at 60 days in old mice compared to young mice. In addition, SDR in old mice resulted in accumulation of IL-10 mRNA and decreased IFN-γ mRNA at 60 days. Also, confocal microscopy of lung sections from old SDR mice showed increased number of CD4 T cells which express LAG3 and CD49b, markers of IL-10 secreting regulatory T cells. Further, we also demonstrated that CD4 T cells from old SDR mice express IL-10. Thus, we conclude that psychological stress in old mice prior to infection, increases differentiation of IL-10 secreting T cells, which over time results in loss of control of the infection.

## Key Points

1. Social Disruption Stress suppresses lung inflammation in old mice

2. SDR prior to infection increases *M.tb* burden at 60 day post-infection in old mice.

3. SDR in old infected mice induces expression of LAG3, CD45b, and IL-10 in CD4 T cells.

## Introduction

The global population of people over age 65 is forecast to increase 3-fold by 2050 (to 1.5 billion), a growth rate that will place extraordinary demands on our health care systems (United Nations, D. o. E. a. S. A., Population Division, 2017). Although increased life expectancy is a major achievement of modern medicine, an equally important concern is quality of life for our elderly. In this regard, aging impacts nearly all organ systems, including the immune system, which exhibits a dramatic functional decline over our life span. In turn, waning or altered immunity renders our elderly populations more susceptible to infections, such as influenza, pneumococcal pneumonia, tuberculosis (TB) and more recently COVID19 (Zevallos and Justman, 2003; Krone et al., 2014; Bahadoran et al., 2016; Mehta and Dutt, 2016; Nikolich-Zugich et al., 2020). A second compounding factor for disease susceptibility and outcomes is psychological stress, which is particularly acute in older individuals, who may suffer from chronic illnesses, and/or have limited financial means, emotional support networks, or mobility. An individual’s response to such unfortunate circumstances ranges from an acute to a sustained chronic stress response, the latter of which results in anxiety, depression, and increased susceptibility to diseases, including infections (Cohen et al., 2007). The mechanisms by which psychological stress compounds age-related pathologies in response to infections are poorly understood. Thus, this study examined the effect of stress on *Mycobacterium tuberculosis* (*M.tb*) infection the causative agent of TB, and a continuing threat to global health.

Individuals who are 50 years of age or older account for over 50% of all TB deaths (Negin et al., 2015). While many elderly TB cases occur after reactivation of a latent infection, older individuals are also more susceptible to developing active TB after a primary infection (Rajagopalan, 2001). These age-associated vulnerabilities primarily stem from senescence of the adaptive immune system, in particular T cell function (Weiskopf et al., 2009; Ferrando-Martinez et al., 2011; Larbi et al., 2011), and elevation of chronic low-grade inflammation (termed inflammaging) (Franceschi et al., 2000). A primary role for inflammaging in age-dependent decline in immune response is provided by studies with mice. Upon *M.tb* infection older C57BL/6 mice (18 months) are able to control the infection better than young mice (3 months), but over time gradually lose control of the infection compared to young mice (Orme, 1995; Turner et al., 2002; Vesosky and Turner, 2005), a phenomenon that has been attributed to inflammaging (Piergallini and Turner, 2018). Recently, we characterized the impact of aging on the phenotype of mouse alveolar macrophages (AMs) and their immune response to *M.tb* (Lafuse et al., 2019). We reported that AMs from old mice expressed higher mRNA levels of pro-inflammatory cytokines and contained higher cytokine levels in the bronchial fluid compared to young mice, which is indicative of inflammaging occurring in the lung. Also, we identified two distinct AM subpopulations, a major CD11c^+^ CD11b^-^ population and a minor CD11c^+^ CD11b^+^ population that is increased 4-fold in old mice. We showed that this minor population expressed a unique inflammatory mRNA signature and enhanced *M.tb* phagocytosis and survival compared to the major population, which expressed a more immune regulatory mRNA signature.

To examine the effects of psychological stress on the immune system we have utilized social disruption stress (SDR), a mouse social stressor model that involves repeated defeat in subordinate mice (Allen et al., 2012a; Allen et al., 2012b; Lafuse et al., 2017). Exposure to SDR increases serum levels of pro-inflammatory cytokines IL-1β and IL-6 (Stark et al., 2002; Engler et al., 2008; Kinsey et al., 2008) and increases spleen mass due to accumulation of CD11b^+^ myeloid cells, which are primed for increased cytokine and microbicidal activity (Stark et al., 2002; Kinsey et al., 2007; Engler et al., 2008; Allen et al., 2012a; Allen et al., 2012b). Other studies have shown that SDR induces translocation of primed monocytes from the spleen into peripheral tissues and enhances inflammation (Curry et al., 2010; McKim et al., 2016; McKim et al., 2018). However, the impact of SDR in response to *M.tb* infection in the context of aging has not been not studied.

The current study examined the effect of SDR on the lungs of young and old mice and the response to infection with *M.tb.* In striking contrast to previous studies, we report that SDR does not enhance inflammation in the lung but induces an immune suppressive environment with decreased pro-inflammatory cytokine levels in the BAL fluid and decreased cytokine mRNA levels in AMs. The greatest effect was observed in stressed old mice. AMs from old mice were not primed and instead produced decreased pro-inflammatory cytokine mRNA when stimulated with a TLR2 ligand. SDR prior to infection in old mice substantially increased the *M.tb* burden at 60 days post infection, indicating that SDR exacerbates the loss of control of *M.tb* that occurs with aging. Analysis of lung mRNA from infected mice indicates at 60 days, SDR inhibited the expression of IFN-γ mRNA and increased expression of the immunosuppressive cytokine IL-10. mRNA levels of JAK3, which is most commonly expressed in T cells, were also increased in the lungs of old SDR mice, suggesting increased numbers of T cells. Confocal microscopic analysis of lung sections from old SDR mice showed increased numbers of CD4 T cells that express LAG3 and CD49b, markers of regulatory T cells (Gagliani et al., 2013). Further, we observed that CD4 T cells in old SDR mice express IL-10. Thus, our study suggests that stress in old mice regulate the differentiation of CD4 T cells from host protective Th1 cells to IL-10 secreting regulatory T cells, which would favor *M.tb* growth and loss of control of the infection at day 60. Also, this study indicates that psychological stress prior to infection can have long-term effects on the immune response to infection in the elderly. This is not only relevant to TB but is likely relevant to other lung infections including SARS-CoV2.

## Materials and methods

### Mice

Male C57BL/6 mice were purchased from Charles River Laboratories (Wilmington, MA) at an age of 3 months (young) or 18 months (old) obtained through a contract to Charles River Laboratories from the National Institute of Aging. Mice were housed in microisolator cages at 3 mice/cage and acclimated to the facility for 1 week prior to use. Male retired CD1 breeder mice were purchased from Charles River Laboratories and housed at 1 mouse/cage. Mice were maintained on a 12 h light/dark schedule and food and water available *ad libitum.* All procedures were approved by The Ohio State University Institutional Laboratory Animal Care and Use Committee.

### Social disruption

The SDR stressor was performed as previously described (Lafuse et al., 2017). SDR involves repeated social defeat from interactions between an aggressive intruder male mouse and resident male mice for six consecutive nights. The C57BL/6 mice were randomly divided into SDR or home cage control mice. A retired CD1 breeder mouse was placed in a cage of three C57BL/6 mice, starting at 3 PM. If the intruder mouse did not initiate an attack within 10 min and defeat all three resident mice, the mouse was removed and replaced with a new intruder mouse. After 2 h, the intruder mouse was removed, and resident mice left undisturbed until the following day when the SDR protocol was repeated. The morning after the sixth cycle of SDR, the mice were sacrificed or transferred to the BSL3 vivarium for infection with *M. tb.* Mice with wounds penetrating the cutaneous layer were not used in the study. Home cage control mice were left undisturbed throughout the experiment.

### Isolation of AMs and bronchial fluid

Young and old SDR and Home Cage control mice were euthanized by CO_2_ following a protocol approved the Ohio State University Institutional Laboratory Animal Care and Use Committee. AMs and bronchial lavage fluid (BAL fluid) were obtained by bronchoalveolar lavage of mice by washing the lungs 10 times with 0.50 ml of sterile endotoxin-free saline (0.90% NaCl). AMs were collected by centrifugation at 300 X g for 10 min. In each experiment, AMs were pooled from 3 SDR or Home Cage mice (~1 million pooled cells). Aliquots of the pooled AMs (each ~50,000 cells) were pelleted, and RNA was isolated using TRIzol reagent (Invitrogen) for basal RNA measurements by quantitative RT-PCR (qRT-PCR). The remaining AMs were used for flow cytometry and TLR2 stimulation experiments. The supernatant fraction containing BAL fluid from individual mice was quickly frozen and stored at -80°C until use.

### Isolation of splenocytes

Spleens were removed and a single-cell suspension was obtained by dicing each spleen into 1-2 mm pieces and gently pressing through a 70 μm cell strainer. Erythrocytes were lysed with Gey’s lysis buffer (8 mM NH_4_Cl, 5KHCO_3_) and splenocytes suspended in PBS.

### Flow cytometry

AMs (1 X 10^5^) and splenocytes (6 X 10^5^) were aliquoted into FACS tubes and centrifuged at 300 X g for 10 min. Cell pellets were resuspended in 100 ul of FACs buffer (PBS with 2% BSA and.10% sodium azide) and incubated with 1 ul Seroblock FcR (BioRAD) for 10 min. Cells were then stained with Abs for 30 min in the dark. Cells were washed twice FACS buffer and analyzed on a LSR II cytometer using FlowJo software (Tree Star). Abs (purchased from Biolegend) used were: BV421 anti-CD11b (clone M1/70), BV785 anti-CD11c (clone N418), BV605 anti-Ly6C (clone HK1-4), allophycocyanin anti-Ly6G (clone 1A8), Alexa 488 anti NK1.1 (clone P136), Alexa 488 anti-CD45R (B220) (clone RA3-6B2), Alexa 488 anti-CD4 (clone GK1.5), and Alexa 488 anti-CD8α (clone 53-6.7). Isotype controls were BV421 rat IgG2b, BV785 hamster IgG, BV605 rat IgG2a, and allophycocyanin hamster IgG. The numbers of each myeloid population/spleen were calculated from the percentage of the population determined by flow cytometry and total spleen counts.

### ELISA

Cytokine levels of BAL fluid were determined by ELISA, according to manufacturer’s instructions. Aliquots of BAL fluid were added to individual ELISA plate wells (Nunc MaxiSorp plates; Thermal Fisher Scientific). Turbo-TMB-ELISA Substrate (Thermal Fisher Scientific was used for detection. ELISA kits were purchased from R&D Systems (CCL2 [DY439], IL-1β [DY421], TNF-α [DY410], IL-12p70 [DY419], GM-CSF [DY415], M-CSF [DY416], IFN-γ [DY485], IL-17 [DY421] or IL-10 (MABTECH 3432). ELISA kit for norepinephrine was obtained from G-Biosciences. Corticosterone ELISA kit was obtained from ENZO. Protein concentrations of BAL fluid were determined by Bradford assay (BioRAD).

### TLR2 stimulation of AMs

AMs were plated in 48-well plates at 50,000 AMs per well in RPMI 1640 media containing 2mM glutamine, 1% pencillin/streptomycin, and 10% heat-inactivated FBS. After 2 h, nonadherent cells were removed by washing and the AMs incubated with 100 ng/ml Pam_3_ CSK4 (InVivogen). After 6 h incubation, RNA was isolated using TRIzol reagent.

### M. tb infection

Young and old SDR and Home Cage mice were infected with *M.tb* H_37_R_v_ (100-200 CFU/mouse) using an Inhalation Exposure System (Glas-col, Terre Haute, IN). After 30 and 60 days of infection, mice were euthanized by CO_2_ and lung lobes removed. The *M.tb* burden was determined by plating serial dilutions of tissue homogenates of the left lung lobe onto OADC-supplemented 7H11 agar. *M.tb* CFUs were counted after 3 weeks at 37°C. Right lung lobes from each mouse were homogenized in TRIzol for RNA analysis and PBS for cytokine analysis by ELISA. Right lung lobes were also fixed in 10% formalin and embedded in paraffin. The lung lobes were sectioned and stained with hematoxylin and eosin by the OSU Comparative Pathology and Mouse Phenotyping Shared Resource for Confocal Microscopy.

### RNA isolation and qRT-PCR

RNA from AM TRIzol lysates was extracted with chloroform and precipitated with isopropanol. The RNA pellet was washed once with 75% ethanol and RNA reconstituted with DNase/RNase-free water. To isolate RNA from *M.tb* infected lung tissues, TRIzol lysates were extracted with chloroform and purified by Qiagen RNAeasy mini columns with on-column DNA digestion. RNA was reverse transcribed using random primers by the Promega Reverse Transcription System (Thermal Fisher Scientific). Expression of mRNA was determined by qRT-PCR of duplicate samples using IQ SYBR Green Supermix (BioRad). The amplification conditions were 95°C for 2 min, followed by 45 cycles of 95° C for 15s, 60°C for 30s, and 72°C for 30s. Validated mouse primers listed on PrimerBank (Wang et al., 2012) were used and sequences listed in **Supplemental Table 1**. Expression of mRNA was calculated by the Δ threshold cycle method using β-actin as the normalizer (Livak and Schmittgen, 2001). Levels of mRNA in AMs and mRNA in lung tissue from *M. tuberculosis* infected mice were expressed relative to the level of β-actin.

### Confocal microscopy of lung sections

Lung sections from *M.tb* infected mice were subjected to deparaffinization and antigen retrieval prior to blocking for 1 h with 10% goat serum and 1% FBS in a humidified chamber (Wu et al., 2019). The sections were incubated at 4°C with primary antibodies against CD4 (rabbit mAb D7D2Z, Cell Signaling), LAG3 (rat mAb C9BZW, BioLegend), CD49b (biotin labeled rat mAb DX5, BioLegend), and IL-10 (rat Mab MT60, MABTECH). After washing PBS were incubated for 1 h with secondary antibodies Alexa 488 labeled donkey F(ab´)_2_ anti-rabbit IgG and Alexa 647 labeled donkey F(ab´)_2_ anti-rat IgG (Abcam) and Alexa-647 labeled streptavidin (BioLegend). Nuclei were stained with DAPI. All fluorescence images were captured using an Olympus FV 1000 Spectral Confocal system. Mean fluorescence intensity of cells was determined using Image J (version 2.0.0).

### Statistical analysis

All data were expressed as mean± SEM. Statistical analysis was performed using GraphPad Prism software. Comparisons were done using one-way ANOVA with Tukey multiple-comparisons *post hoc* test. Differences were considered statistically significant when p values were *p <*0.05.

## Results

### Effect of SDR on myeloid populations in the spleen and AM populations in the lung

Our previous studies have shown that SDR in young mice results in the accumulation of CD11b^+^ myeloid cells in the spleen that express higher levels of IL-1β mRNA than Home Cage control mice (Lafuse et al., 2017). SDR in old mice also induced accumulation of CD11b^+^ myeloid cells as evidenced by

increased spleen weight (**Supplemental Figure 1A**) and increased numbers of CD11b^+^ Ly6C^+^ monocytes and CD11b^+^ Ly6G^+^ neutrophils in old mice (**Supplemental Figures 1B–D**). This indicates that the response of old mice to SDR in the spleen is identical to the response of SDR in young mice.

**Figure 1:**
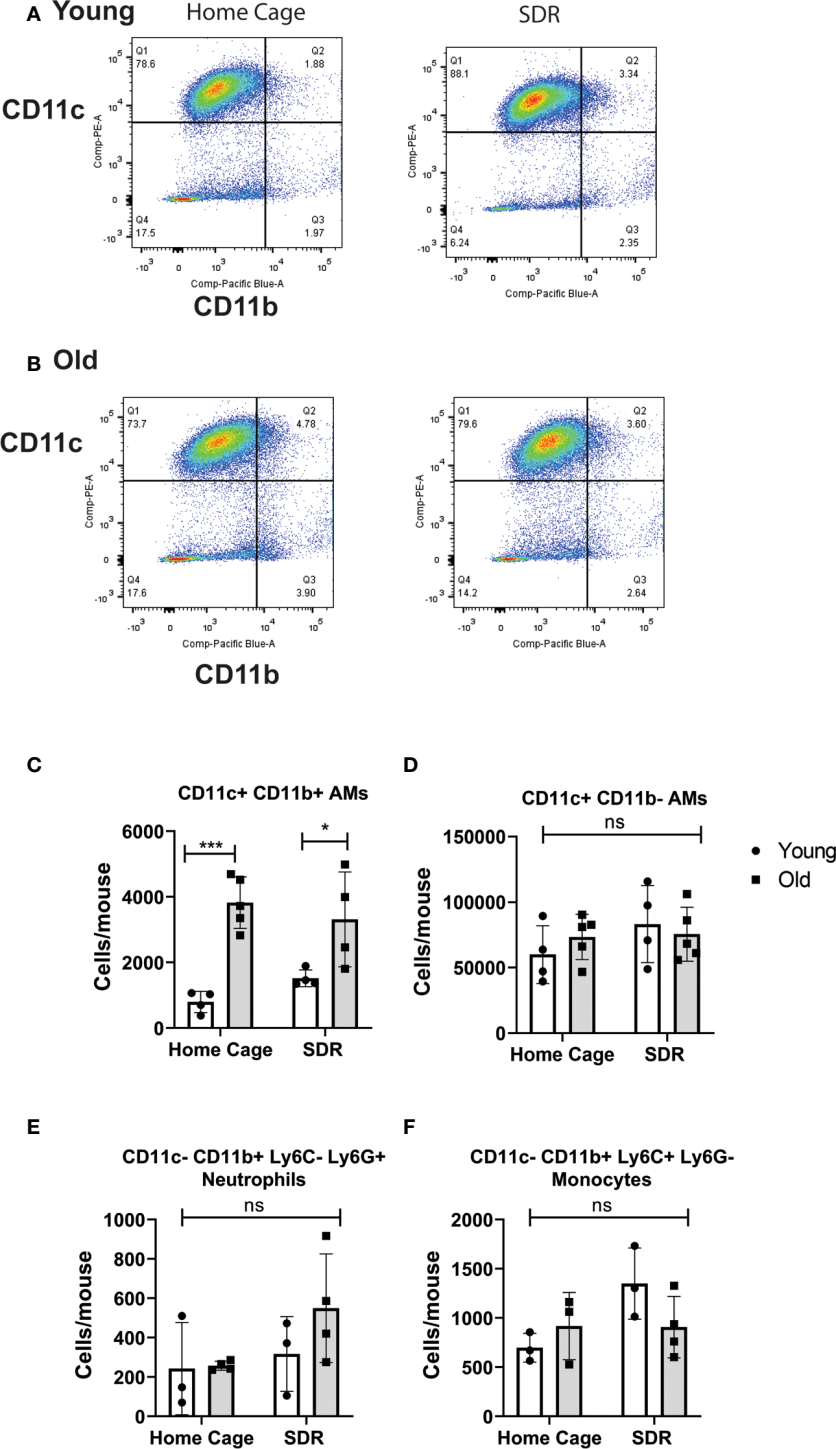
Flow cytometry analysis of myeloid cells in BAL fluid from Home Cage and SDR mice. CD11c/CD11b flow plots of BAL cells from young **(A)** and old **(B)** mice. CD11c^+^ CD11b^-^ AMs are in quadrant 1. CD11c^+^ CD11b^+^ AMs are in quadrant 2. Monocytes and neutrophils present in the CD11c^-^ CD11b^+^ cells (quadrant 4) were detected using antibodies to Ly6G and Ly6C. Cell numbers of each population present in BAL fluid were determined. **(C)** CD11c^+^ CD11b^-^ AM population **(D)** CD11c^+^ CD11b^+^ AM population **(E)** Neutrophils **(F)** Monocytes. Each symbol represents cells present in individual mice. Statistical analysis was performed by one-way ANOVA with Tukey multiple-comparisons *post hoc* test. N=4-5 mice/group. Data are shown as mean ± SE. *p <0.05, ^***^ *p <* 0.001. ns, non significant.

Studies have shown that SDR induces migration of primed monocytes from the spleen into various organs including the lung (Curry et al., 2010; McKim et al., 2016; McKim et al., 2018). Thus, we next examined the effect of SDR on the AM populations in the lung alveolar space. We have identified two AM populations, a major CD11c^+^ CD11b^-^ population with a regulatory phenotype and a minor CD11c^+^ CD11b^+^ inflammatory population that is increased 4-fold in old mice (Lafuse et al., 2019). AMs from old mice expressed CD64 (FcγR1), which is absent in dendritic cells (Immunological Genome Consortium, 2012), suggesting that these cells are not dendritic cells. Sorted CD11c^+^ CD11b^+^ AMs from old mice expressed higher mRNA levels of CCL2, IL-1β, and IL-6, whereas CD11c^+^ CD11b^-^ AMs expressed higher levels of immune-regulatory IFN-β, and IL-10. The CD11c^+^ CD11b^+^ population also showed enhanced *M.tb* phagocytosis and survival compared to the CD11c^+^ CD11b^-^ population. Thus, this study identified a major AM population with immune-regulatory function and a minor AM population with an inflammatory signature and is more permissive for *M.tb.* In the current study, we determined whether SDR altered these AM populations in the lung. AMs were isolated from the BAL fluid of young and old mice and the two AM populations analyzed by flow cytometry. The dot plots shown in **Figures 1A, B** is the gating strategy of BAL cells with different macrophage markers (CD11b and CD11c). As we previously reported there were more CD11c^+^ CD11b^+^ AMs in the old mice (**Figure 1C**). However, SDR did not affect the numbers of the two AM populations (**Figures 1C, D**). We noted the presence of cells that were CD11c^-^ CD11b^+^, which are Ly6G^+^ neutrophils and Ly6C^+^ monocytes. The numbers of neutrophils and monocytes present in the BAL fluid were not significantly different in young and old mice and was not altered by SDR (**Figures 1E, F**). A previous study (Curry et al., 2010) reported that CD11b^+^ myeloid cells migrate into the lung in response to SDR. However, our studies of BAL fluid myeloid cells suggest that CD11b^+^ myeloid cells that migrate into the lung in response to SDR do not accumulate in the alveolar space and are likely retained in the lung interstitium.

### SDR induces an immune suppressive environment in the lung of aged mice

**Figure 2:**
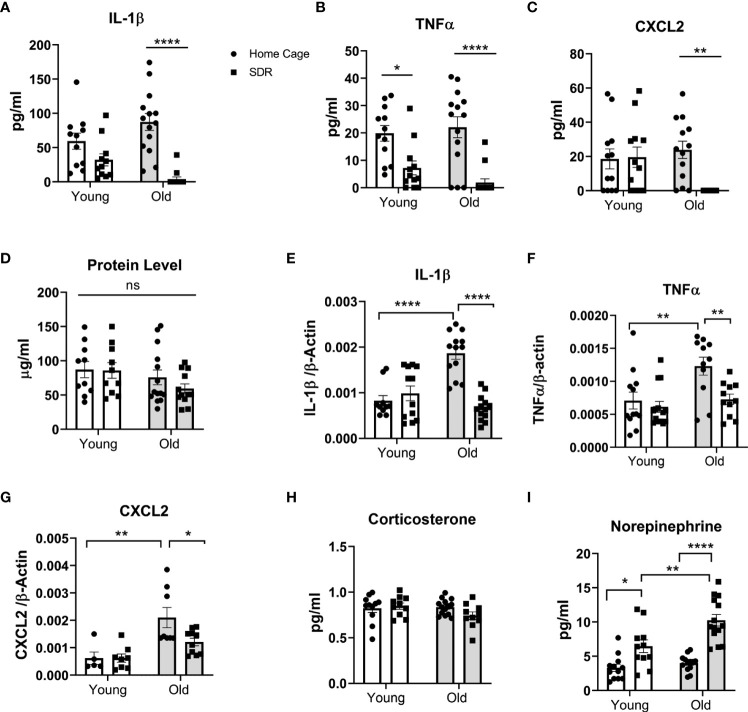
SDR in old mice suppresses cytokine levels in BAL fluid and basal mRNA levels in alveolar macrophages. BAL fluid and alveolar macrophages were isolated from individual young (3 months) and old (18 months) Home Cage mice and mice subjected to 6 cycles of SDR. **(A–C)** Cytokine levels in BAL fluid determined by ELISA. Protein levels in BAL fluid were determined by Bio-RAD protein assay **(D)**. **(E–G)** Basal cytokine mRNA expression in AMs. RNA was isolated from individual mice without culture and mRNA levels determined by qRT-PCR. Expression levels were normalized to β-actin mRNA. Protein levels in BAL fluid were determined by Bio-RAD protein assay. **(H, I)** Corticosterone and Norepinephrine levels in BAL fluid determined by ELISA. Each symbol represents individual mice. N=9-15 mice/group. Data are shown as mean ± SE. Statistical analysis was performed by one-way ANOVA with Tukey multiple-comparisons *post hoc* test. ns, non significant. ^*^ *p* < 0.05, ^**^ *p <* 0.01, ****p < 0.0001.

All previous studies have indicated that SDR enhances inflammation in tissues. Thus, to test whether this also holds true for the lung of aged mice, we measured cytokine levels in BAL fluid and cytokine mRNA levels in AMs. Surprisingly, SDR induced lower basal levels of pro-inflammatory cytokines IL-β and TNFα and the CXCL2 chemokine in BAL fluid of old and young mice, with the greatest effect in old mice (**Figures 2A–C**). Particularly noteworthy was that SDR in old mice greatly attenuated the production of IL-1β and TNFα, as well as the chemokine CXCL2. We also assayed for IL-12p40, CCL2, and IL-6. However, the levels of these cytokines were at or below the detection level. We also determined total protein levels in BAL fluid and found no significant differences in protein levels with age or SDR (**Figure 2D**).

To determine the effect of SDR on mRNA expression levels of various cytokines and chemokines in the AMs of young and old mice, we isolated RNA from AMs without culture and the mRNA levels of different cytokines and chemokines were determined by qRT-PCR. Our data indicate that cytokine and chemokine mRNA levels paralleled the cytokine levels in the BAL fluid with decreased levels of IL-1β, TNFα, and CXCL2 mRNA in old SDR mice (**Figures 2E–G**). SDR in young mice had no effect on cytokine mRNA levels. We have previously shown that inflammaging significantly enhances the expression of pro-inflammatory cytokines (Lafuse et al., 2019). Consistent with the previous study, mRNA levels of IL-1β, TNFα, and CXCL2 were significantly higher in old Home Cage mice compared to young Home Cage mice (**Figures 2E–G**). However, we report that SDR stress significantly mitigated the inflammaging mediated upregulation of cytokines and chemokines. Together, these studies indicate that SDR in aged mice alters the environment of the lung to one that is more immunosuppressive or to one that more closely resembles the environment in young mice.

### SDR increases levels of norepinephrine in BAL fluid

Previous reports indicate that SDR stress increases corticosterone and norepinephrine (NE) in the blood (Avitsur et al., 2001; Hanke et al., 2012). As such, we determined if SDR also increases corticosterone and norepinephrine in the BAL fluid. As shown in **Figure 2H**, we found no significant differences in corticosterone levels in BAL fluid from young and old mice exposed to SDR. However, SDR increased NE levels in both and young mice with higher levels in old mice (**Figure 2I**). This suggests that the accumulation of locally produced NE in the alveolar space of the lung may contribute to the immune-suppressive environment induced in the lung by SDR.

### AMs from old mice are not primed but immune suppressed in response to TLR2 stimulation

**Figure 3:**
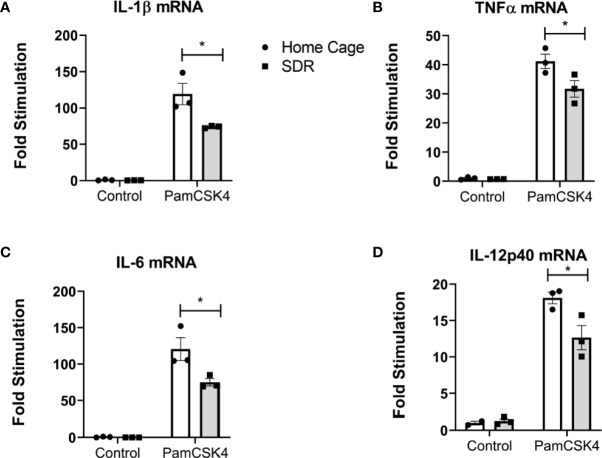
SDR suppresses TLR2 induced pro-inflammatory cytokine mRNA levels in AMs from old mice. AMs were pooled from six old Home Cage and six SDR mice were cultured at 37° for 2 hrs. Nonadherent cells were removed by washing and adherent cells stimulated with TLR2 ligand Pam_3_CSK_4_ (100 ng/ml) for 6 hrs. RNA was isolated and cytokine mRNA levels determined by qRT-PCR. **(A-D)** RNA levels are expressed relative to β-actin mRNA levels. Results are representative of two independent experiments with 3 wells/treatment. Data are shown as mean ± SE. Statistical analysis was performed by one-way ANOVA with Tukey multiple-comparisons *post hoc* test. ^*^ *p* < 0.05.

In our previous studies (Allen et al., 2012a; Allen et al., 2012b; Lafuse et al., 2017) we demonstrated that SDR primes monocytes to respond with increased production of pro-inflammatory cytokines when stimulated with a TLR agonist. To determine whether SDR stress alters the TLR mediated immune response of AMs from old mice, we harvested AMs from old Home Cage control mice and SDR mice and then stimulated with the TLR2 agonist Pam_3_ CSK4. RNA was isolated after 6 h and the mRNA expression levels of various cytokines determined by qRT-PCR. Instead of being primed, the mRNA levels of pro-inflammatory cytokines IL-1β, TNFα, IL-12p40, IL-6 were suppressed by SDR stress (**Figures 3A–D**). This indicates that the immune suppressive environment induced by SDR in old mice inhibits the ability of AMs to respond to TLR2 with a pro-inflammatory cytokine response.

### SDR stress exacerbates M.tb growth in the lungs of old mice and reduces the formation of pneumonia

**Figure 4:**
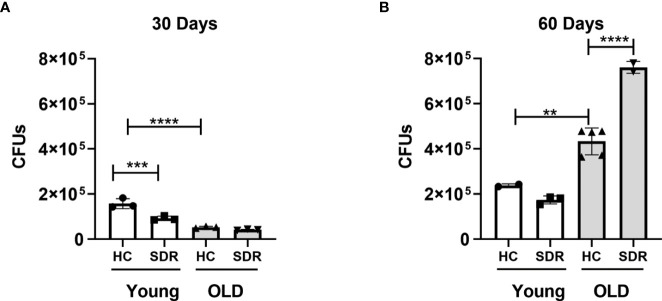
Old mice initially control *M.tb* infection better than young mice but eventually lose control of *M.tb* infection, which is further exacerbated by SDR. Young and old Home Cage mice subjected to six cycles of SDR were infected with low dose aerosol of *M. tuberculosis*. At 30 days **(A)** and 60 days **(B)**, the left lung lobes were homogenized and *M. tuberculosis* CFUs enumerated by plating on 7H11 duplicate plates. Results are representative of two independent experiments with 3 mice/group per time point. Data are shown as mean ± SE. Statistical analysis was performed by one-way ANOVA with Tukey multiple-comparisons *post hoc* test. **p < 0.01, ^***^ *p* < 0.001, ****p < 0.0001.

Since we found that SDR stress inhibits the inflammatory response in AMs, we next initiated studies to investigate the effects of SDR on *M.tb* infection. Young and old mice were subjected to 6 cycles of SDR and infected with *M.tb* for 30 and 60 days (30 days is early in the adaptive immune response and 60 days is later in the response). At each time point, mice were euthanized, and lungs were harvested. The left lung lobes were used for CFU assay. Right lung lobes were used for RNA isolation and lung sections for confocal microscopy. Previous studies have shown old mice exhibit early control of *M.tb* that is sustained through 21 days and then gradually wanes (Cooper et al., 1995; Turner and Orme, 2004; Vesosky and Turner, 2005; Vesosky et al., 2006; Rottinghaus et al., 2009; Vesosky et al., 2009). Consistent with these studies, old Home Cage mice at 30 days post-infection controlled the infection better than young Home Cage mice (**Figure 4A**). SDR had no effect in old mice at 30 days post infection, but interestingly, SDR in young mice decreased *M.tb* burden. However, the early control in old mice is transient (Orme, 1995; Turner et al., 2002; Vesosky and Turner, 2005). Thus, at 60 days of infection, there were more *M.tb* growth in the lungs of old Home Cage compared to young Home Cage mice (**Figure 4B**). Furthermore, SDR in old mice significantly increased the *M.tb* burden, while not affecting the *M.tb* burden in young mice. In contrast, at 60 days of infection in young mice there was only a slight increase in CFUs compared to CFUs at 30 days, which suggests the infection in young mice has reached a plateau level by 60 days. In a repeat experiment (**Supplemental Figure 2**), *M.tb* infection in young mice also reached a plateau level of infection by 60 days, while the *M.tb* burden in old mice increased at 60 days post infection and the highest level of infection was in the old SDR mice. Together, the two experiments indicate that old mice have reduced ability to control the infection long-term and exposure to SDR prior to infection in old mice exacerbates the loss of control that occurs with aging.

Lung sections from the infected mice were stained with hematoxylin and eosin. Representative images are shown in **Supplemental Figure 3**. In young and old home cage and SDR mice infected with *M.tb*, the images shows areas of pneumonia at day 30 and 60 post infection. However, in old SDR mice the areas of pneumonia are smaller at 30 and 60 days compared to old home cage mice. There are also more lymphocytes surrounding the blood vessels. This suggests that while lymphocytes are able to enter the infected lung of old SDR mice, but they are not able to form pneumonia.

### Old SDR mice express decreased mRNA levels of IFN-γ and increased mRNA levels of IL-10

**Figure 5:**
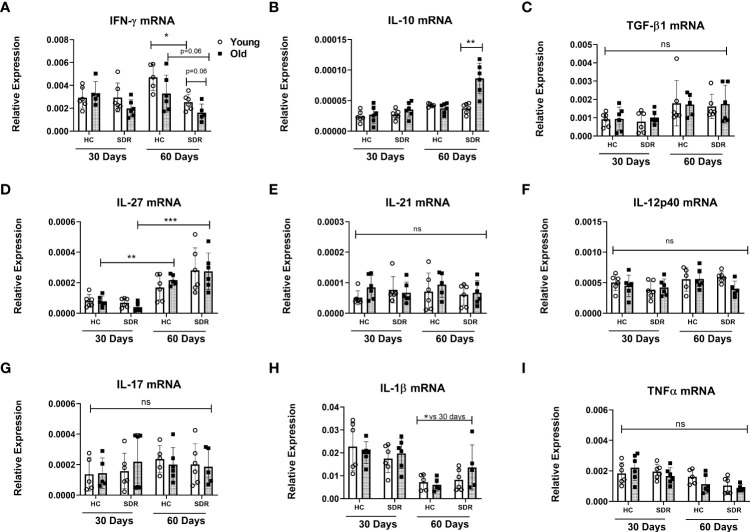
mRNA levels of cytokines genes in the lungs of mice infected with *M.tb.* Young and old Home Cage mice and mice subjected to six cycles of SDR were infected with low dose aerosol of *M. tuberculosis* for 30 and 60 days. RNA was isolated from a right lung lobe and mRNA levels determined by qRT-PCR **(A-I)**. RNA levels are expressed relative to β-actin. Results are pooled from two independent experiments. Each symbol represents individual mice. Data are shown as mean ± SE. Statistical analysis was performed by one-way ANOVA with Tukey multiple-comparisons *post hoc* test. ^*^ *p*< 0.05, ^**^ *p* < 0.01, ***p < 0.001. ns, non significant.

We next examined the mRNA levels of cytokines in the lungs of *M.tb* infected mice. At 30 days post-infection, there were no significant differences in the expression of IFN-γ mRNA (**Figure 5A**). However, at 60 days post infection, old mice had significantly less IFN-γ mRNA, and the lowest levels were observed in the stressed old mice. The stressed old mice also expressed significantly higher levels of IL-10 mRNA (**Figure 5B**). mRNA expression of TGF-β1 did not vary with age or stress (**Figure 5C**). mRNA expression of TGF1-β3 was comparable to the levels of TGF-β1 and also did vary with age or stress (data not shown). We also measured the levels of IL-27, a member of the IL-12 family involved in induction of IL-10-producing T cells (Tait Wojno et al., 2019). IL-27 mRNA was expressed at low levels at 30 days post-infection and at significantly higher levels at 60 days (**Figure 5D**). However, aging or stress had no effect on the levels of IL-27 mRNA. The increase in IL-27 mRNA at day 60 is of interest, since although IL-27 *in vitro* augments differentiation of naïve T cells toward a IFN-γ-producing phenotype (Takeda et al., 2003; Kamiya et al., 2004; Owaki et al., 2006), expression of IL-27 *in vivo* during various infections, including *M.tb*, has been shown to have a detrimental effect on the control of infections and reduces the amount of IFN-γ produced by CD4 T cells (Abdalla et al., 2015; Wang and Liu, 2016). There were no significant differences in the IFN-γ inducing cytokines IL-12p40 (**Figure 5F**) and IL-12p35 (data not shown) mRNA levels between home cage and SDR and also between 30 and 60 days. This suggests that lack of IL-12 at 60 days post infection is not a factor in the decreased IFN-γ mRNA. There were also no significant differences in the mRNA levels of T cell cytokines IL-21 (**Figure 5E**) and IL-17 (**Figure 5G**). The pro-inflammatory cytokines IL-1β (**Figure 5H**) and TNFα (**Figure 5I**) were expressed at high levels, but there was no effect of aging and stress on the mRNA levels. Interestingly, IL-1β mRNA levels were significantly lower at 60 days post infection compared to 30 days post infection. Together, these data show that SDR in old mice increased expression of IL-10 mRNA and decreased expression of IFN-γ mRNA at 60 days post infection, which is consistent with the loss of control of the *M.tb* infection in these mice.

### Old SDR mice express higher mRNA levels of JAK3 but the mRNA levels of transcription factors that define T cell subsets are not altered by age or stress

**Figure 6:**
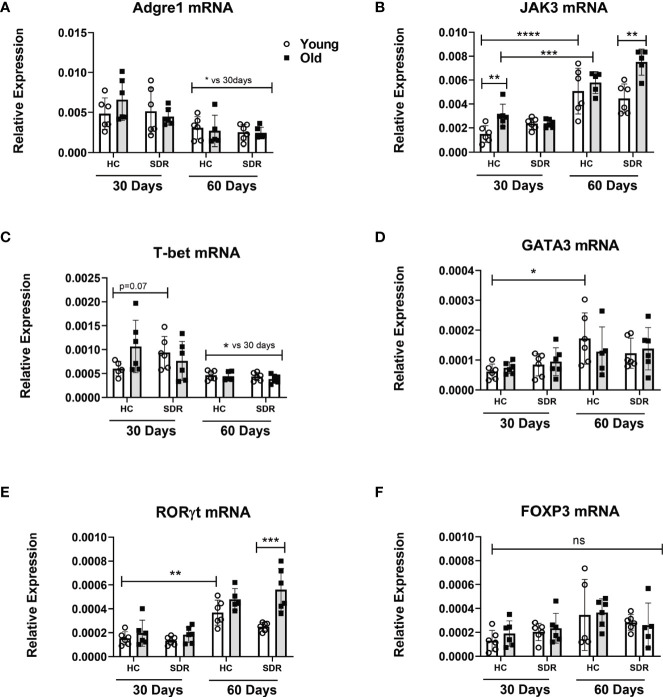
mRNA levels of macrophage and T cell genes in the lungs of mice infected with *M.tb.* Young and old Home Cage mice and mice subjected to six cycles of SDR were infected with low dose aerosol of *M. tuberculosis* for 30 and 60 days. RNA was isolated from a right lung lobe and mRNA levels **(A–F)** determined by qRT-PCR. RNA levels are expressed relative to β-actin. Each symbol represents individual mice. Data are shown as mean ± SE. Statistical analysis was performed by one-way ANOVA with Tukey multiple-comparisons *post hoc* test. Results represent 3 mice/group per time point. Data are shown as mean ± SE. ^*^ *p*< 0.05, ^**^ *p* < 0.01, ***p < 0.001, ****p < 0.0001. ns, non significant.

Granuloma formation in *M.tb* infections involves the migration of macrophages and T cells into the infected lung. Therefore, we determined whether aging and SDR stress influenced mRNA levels of constitutively expressed macrophage and T cell markers in the lungs of *M.tb* infected mice at 30 and 60 days. A widely used marker of macrophages is F4/80, which is coded by the *Adgre1* gene (Waddell et al., 2018). Thus, we investigated the effects of aging and SDR stress on Adgre1 mRNA expression. Analysis of Adgre1 mRNA expression showed that aging or stress did not affect expression of this gene (**Figure 6A**). Interestingly, expression was lower at day 60 compared to day 30. Establishment of protective immunity to *M.tb* infection is dependent on the presence of IFN- γ secreting CD4^+^ T cells (Green et al., 2013). Thus, we determined the effect of aging and SDR stress on CD4^+^ T cells in the lungs of *M.tb* infected mice, we measured the mRNA levels of genes involved in T cell differentiation in young and old mice with and without SDR stress. We first determined the expression of JAK3, which is primarily expressed by T cells and NK cells. At 30 days post- infection, old Home Cage mice expressed significantly higher levels of JAK3 mRNA compared to young Home Cage mice (**Figure 6B**). The expression of JAK3mRNA was not altered by SDR stress in mice at 30 days post infection. However, expression of JAK3mRNA was higher at day 60 compared to day 30 and SDR stress significantly enhanced levels in old mice at 60 days (**Figure 6B**.). This suggests that there are more T cells present at 60 days post infection than 30 days. However, this increase in T cells did not translate into increased IFN-*γ* mRNA levels at 60 days (**Figure 5A**). Nor was there any difference with aging or stress in the mRNA expression of the Th1 transcription factor T-bet at 60 days post infection (**Figure 6C**). In fact, the levels of T-bet mRNA in the lungs were very low, suggesting that T cells present at 60 days post-infection are not Th1 cells. In contrast, at 30 days post infection T-bet mRNA levels were higher, which is consistent with the role of IFN-γ producing Th1 in the early control of infection in old mice (Turner and Orme, 2004; Vesosky et al., 2006; Rottinghaus et al., 2009; Vesosky et al., 2009). Interestingly, SDR stress in young mice had somewhat higher expression than young home cage mice (p= 0.07), which is consistent with the lower *M.tb* infection in these mice (**Figure 4A**). We also measured the mRNA levels of the Th2 transcription factor GATA3 and the Th17 transcription factor RORγt. Aging and SDR had no significant effect on GATA3 mRNA expression, which was expressed at low levels at both 30 and 60 days (**Figure 6D**). RORγt expression was low at both 30 and 60 days, but at 60 days there was significantly higher levels in old SDR mice (**Figure 6E**). Expression of the mRNA levels of the Treg transcription factor FOXP3 in the lungs of infected mice was also low and not affected by age or stress (**Figure 6F**). While increased expression of JAK3 suggests increased T cells at day 60, the data indicates that the increased T cells were not Th Cells or Tregs.

### Expression of LAG3 and CD49b in CD4^+^T cells is upregulated by SDR in old mice

**Figure 7:**
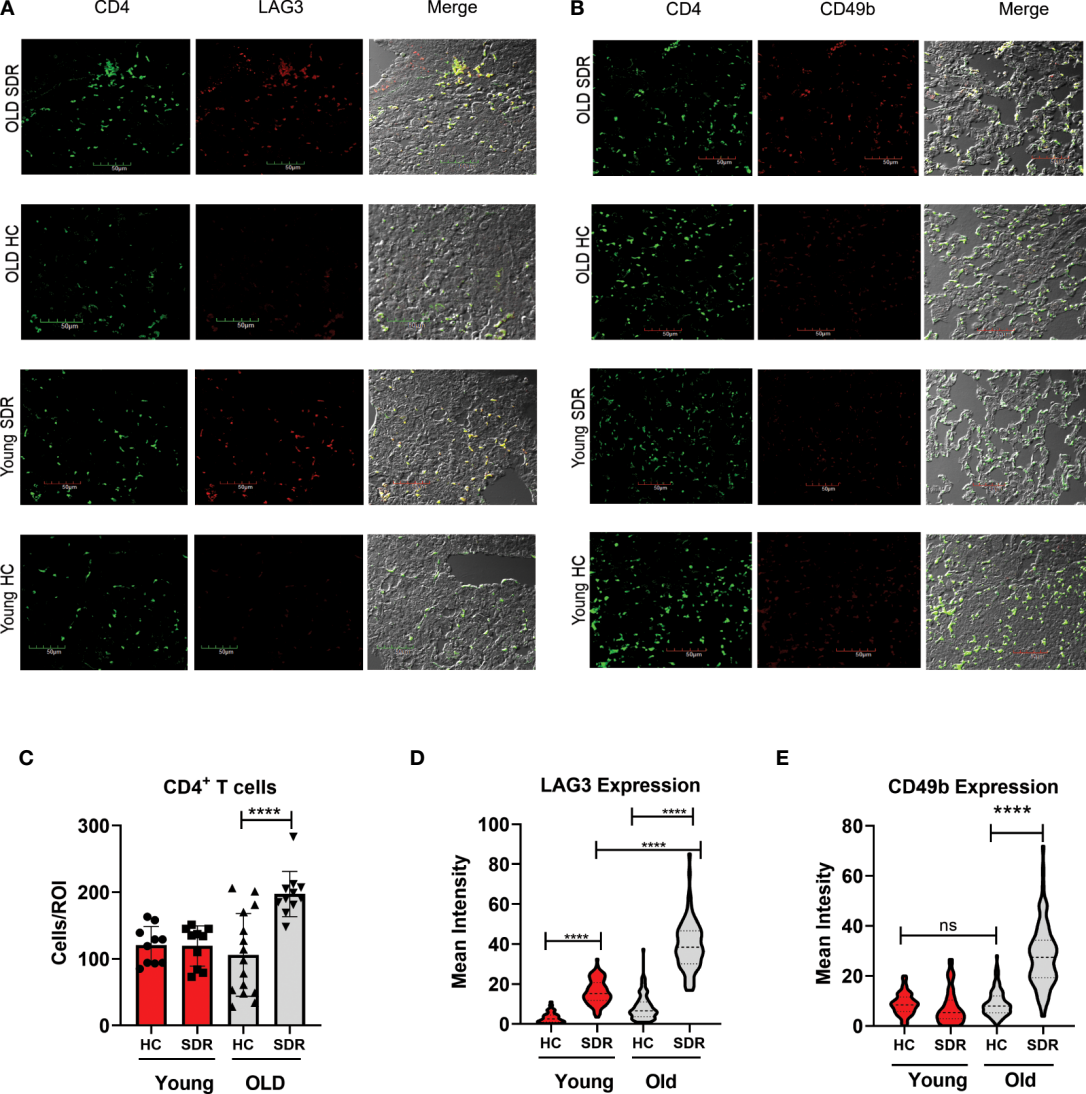
SDR in old mice up-regulate expression of LAG3 and CD49b in CD4^+^T cells **(A)** Representative images of LAG3 expression in CD4^+^ T cells present in regions of pneumonia **(B)** Representative images of CD49b in CD4^+^ T cells. **(C)** Granulomatous tissue in old SDR mice contain more CD4 T cells. CD4 T cells were counted in regions of granulomatous tissue. Each symbol represents a region of granulomatous tissue. N=15 regions/group. Mean fluorescence intensity of LAG3 **(D)** and CD49b **(E)** expression in CD4 T cells was determined using Image **(J)** N= 90 cells/group. Statistical analysis was performed by one-way ANOVA with Tukey multiple-comparisons *post hoc* test. ****p < 0.0001. ns, non significant.

Since our data indicates at 60 days post-infection in old SDR mice there is increased IL-10 mRNA levels in the lungs, we next examined whether CD4^+^ T cells in the lungs of these mice have the phenotype of IL-10 producing regulatory T cells. We examined the CD4^+^ T cell expression of LAG3 and CD49b by confocal microscopy of tissue sections from mice infected with *M.tb* for 60 days. Co-expression of LAG3 and CD49b identifies FOXP3^-^ Type 1 regulatory T cells that produce large amounts of IL-10 (Gagliani et al., 2013). We first determined the numbers of CD4^+^ T cells in the regions of granulomas lung tissue. The numbers of CD4^+^ T cells were significantly increased in old SDR mice (**Figure 7C**), confirming the increased expression of JAK3 RNA in old SDR mice (**Figure 6**). The majority of CD4^+^ T cells in the lungs of old SDR mice expressed both LAG3 (**Figure 7A**) and CD49b (**Figure 7B**). The mean fluorescence intensity of LAG3 expression in CD4^+^ T cells was highly increased by SDR in old mice (**Figure 7D**). LAG3 expression was also increased by SDR in young mice, but the intensity was much less compared to the old mice. The mean fluorescence intensity of CD49b was also highly increased by SDR in old mice (**Figure 7E**), but SDR in young mice had no effect on the expression of CD49b. Thus, the confocal microscopy indicates that SDR prior to infection of old mice results in the presence of CD4 T cells in the lung at 60 days expressing markers of IL-10 secreting regulatory cells.

### CD4^+^T cells in M.tb infected Old SDR mice are producing IL-10

**Figure 8:**
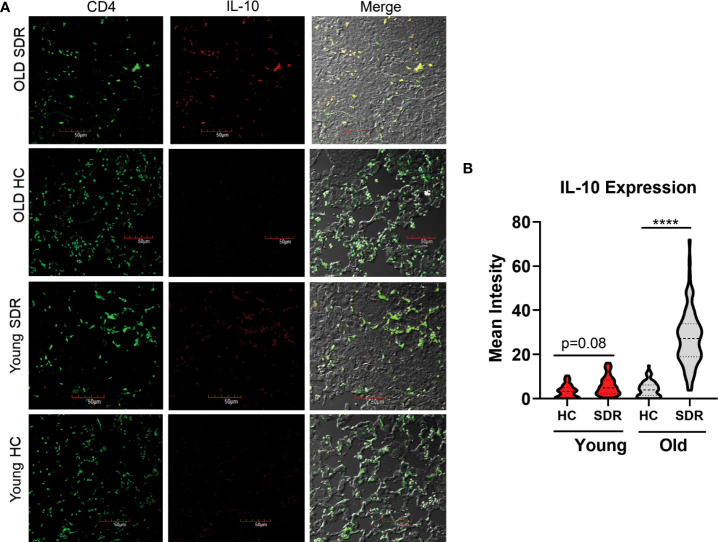
SDR in old mice results in production of IL-10 by CD4^+^ T cells in the lungs of *M.tb* infected mice. Lung tissues sections from mice infected with *M.tb* for 60 days were examined by confocal immunofluorescence microscopy for expression of IL-10 (red fluorescence) in CD4^+^ T cells (green fluorescence) present in areas of pneumonia. **(A)** Representative images of IL-10 expression in CD4^+^ T cells. **(B)** Mean fluorescence of IL-10 expression in CD4 T cells was determined using Image **(J)** N= 90 cells/group. Statistical analysis was performed by one-way ANOVA with Tukey multiple-comparisons *post hoc* test. ****p < 0.0001.

To answer the question whether CD4 T cells in old SDR mice infected with *M.tb* for 60 days are producing IL-10, we performed confocal microscopy of the 60 day lung sections with a monoclonal antibody against mouse IL-10 (**Figure 8A**). Only old SDR mice had significant production of IL-10 (**Figure 8B**) as there was little IL-10 production in old home cage mice or in the young home cage. The mean fluorescence intensity was slightly elevated in young SDR mice, but not to the level of significance. Also, in the old SDR mice the IL-10 producing cells were almost entirely CD4^+^ T cells.

## Discussion

Social disruption stress is a mouse social stressor that has been widely used to study the effects of psychological stress on immune function and behavior. SDR involves both a physical and a psychological component that includes anxiety behavior. This anxiety behavior in the SDR mouse model is similar to anxiety-like behavior that occurs in humans during stressful periods, which is often associated with an enhanced immune responses (Dhabhar, 2014). SDR in mice is also associated with enhanced immune responses. For example, SDR in young adult mice increases the number of splenic CD11b^+^ myeloid cells including monocytes and neutrophils (Kinsey et al., 2007; Allen et al., 2012a; Allen et al., 2012b; Lafuse et al., 2017) by a sympathetic nervous system-induced β-adrenergic signaling pathway initiated by release of catecholamines (Hanke et al., 2012). We showed that the splenic CD11b^+^ myeloid cells are primed by SDR stress induced translocation of gut microbiota to the spleen (Allen et al., 2012a; Lafuse et al., 2017). In the current study, we subjected young and old mice to SDR stress. We first confirmed that SDR stress in old mice also induced splenomegaly and increased numbers of spleen monocytes and neutrophils. This suggests that like young mice, SDR stress in old mice activates the sympathetic nervous system (SNS), resulting in increased spleen monocytes. SDR activation of the SNS has also been shown to enhance inflammation by inducing the translocation of primed monocytes from the spleen into peripheral tissues, including the lung (Curry et al., 2010). However, our study found that SDR stress in young and old mice did not increase CD11c^-^ CD11b^+^ monocytes and neutrophils in the BAL fluid. This indicates that monocytes migrating to lung in response to SDR stress have not migrated into the alveolar space and most likely localize in the lung interstitium. Since SDR stress in young mice at day 30 has lower *M.tb* burden, translocation of primed macrophages induced by SDR may contribute to *M.tb* resistance in young mice. Another possible explanation is that CD4^+^ T cells express the β2-adrenergic receptor and are able to response to norepinephrine (Kohm and Sanders, 2001). *In vitro s*tudies have shown that binding of norepinephrine to naïve CD4^+^ T cells to secrete 2-4 fold more IFN-γ (Swanson et al., 2001). Further, blocking the release of norepinephrine with the terminal adrenergic toxin 6-0H dopamine at time of *M.tb* infection in mice resulted in significant increase in bacterial load in the lungs, while treatment of mice with a β2-agonist decreased bacteria load (Barrios-Payan J. et al., 2016). Thus, SDR in young mice may increase protection against *M.tb* by two possible mechanisms, first, increasing migration of primed macrophages that resist *M,tb* growth and secondly increasing the production of IFN-γ by CD4^+^ T cells *via* norepinephrine. These possible explanations will be explored in future research.

In this study we show that instead of creating an inflammatory environment in the lung alveolar space, SDR stress induces an immuno-suppressive environment with markedly decreased cytokine and chemokine levels in old mice. AMs from old SDR mice also contained less cytokine and chemokine mRNA levels than old Home Cage control mice and produced less cytokine mRNA when stimulated with a TLR2 ligand. We found that SDR stress increased norepinephrine in BAL fluid in both young and old mice, but the levels were higher in old SDR mice. SDR stress in young mice has previously been shown to increase norepinephrine in the blood (Avitsur et al., 2001; Hanke et al., 2012), which represent spill-over from SNS activation in tissues. Thus, the increased levels of norepinephrine in the BAL fluid indicates that SDR stress induces SNS activation in the lung, which results in production of norepinephrine, some of which is retained in the alveolar fluid. Norepinephrine, acting through the β2-adrenergic receptor, suppresses the production of pro-inflammatory cytokines IL-1β, TNFα, IL-6, and IL-12p40 by macrophages and dendritic cells induced by LPS (Koff et al., 1986; Hu et al., 2012; Grailer et al., 2014; Agac et al., 2018). Thus, we conclude that SDR stress induced norepinephrine is contributing factor to the immune-suppressive environment in old mice.

In this study we also examined the effects of psychological stress on *M.tb* infection. Young and old mice were subjected to 6 cycles of SDR prior to infection with *M.tb* for 30 and 60 days. Prior studies have shown that early in the infection old mice control *M.tb* better than young mice but as the infection progresses the old mice lose control of the infection (Orme, 1995; Turner et al., 2002; Vesosky and Turner, 2005). Our studies confirm these previous studies in that we found that at 30 post infection that the lungs of old Home Cage control mice contained fewer *M.tb* CFUs than young Home Cage mice. By 60 days post infection the CFUs were higher in old Home Cage mice than in young Home Cage mice. SDR stress did not have an appreciable effect at 30 days, but at 60 days CFUs were significantly higher in old SDR mice compared to old Home Cage mice. Thus, psychological stress exacerbates the loss of control that occurs with aging.

We also found mRNA levels of IFN-γ were decreased in old SDR mice, while the expression of IL-10 was increased. The expression of the T cell gene Jak3 was also substantially higher at 60 days post infection in old SDR mice compared to old Home Cage mice, suggesting accumulation of T cells in the lungs of old SDR mice. This did not translate into increased IFN-γ and T-bet mRNA, suggesting that the accumulated T lymphocytes in the lungs of old SDR mice are not IFN-γ secreting Th1 cells. Our confocal microscopy confirmed increased numbers of CD4 T cells and showed that the expression of LAG3 and CD49b are upregulated in these cells. Further we found that the CD4 T cells in old SDR mice express IL-10. While IL-10 can be produced by both macrophages and T cells, IL-10 production by macrophages during *M.tb* infection occurs early in the infection, but later production of IL-10 by T cells predominates (Moreira-Teixeira et al., 2017). Our studies show that the predominant cells producing IL-10 in lungs of old SDR is CD4 T cells that express LAG3 and CD49b.

IL-10 suppresses both the innate and adaptive immune responses (Rutz and Ouyang, 2016). IL10 acts on macrophages to inhibit release of pro-inflammatory cytokines including TNF-α, IL-1β, and IL-6 (de Waal Malefyt et al., 1991; Fiorentino et al., 1991). IL-10 inhibits antigen presentation by down-regulating expression of MHC II and co-stimulator CD86 on antigen presenting cells and inhibits production of IL-12 required for CD4 Th1 differentiation (de Waal Malefyt et al., 1991; D’Andrea et al., 1993; Willems et al., 1994; Creery et al., 1996). IL-10 can also act on T cells to inhibit proliferation and cytokine production (de Waal Malefyt et al., 1993; Groux et al., 1996; Rojas et al., 1999). Previous reports have indicated that IL-10 plays an inhibitory role in controlling *M.tb* infections in mice (Turner et al., 2002; Beamer et al., 2008; Cyktor et al., 2013). Interestingly, similar to our study, transgenic C57BL/6 mice producing IL-10 under the control of the IL-2 promoter are able to control *M.tb* infection during the early stage, but during the chronic stage there was a significant increase in bacterial load in the lung (Turner et al., 2002). Disease progression in *M.tb* susceptible strain CBA/J also correlates with high levels of IL-10 in the lung (Turner et al., 2002). Blocking IL-10 in CBA/J mice during the chronic stage of infection improved control of bacterial load and improved survival (Beamer et al., 2008). Further, CBA/J mice deficient in IL-10 were able to contain the *M.tb* infection and formed mature, fibrotic granulomas (Cyktor et al., 2013).

IL-10 is produced by CD4^+^ FOXP3^-^ T regulatory type I (Tr1) cells and CD4^+^ CD25^+^ FOXP3^+^ Treg cells (Roncarolo et al., 2006; Wei et al., 2017; Roncarolo et al., 2018). Co-expression of LAG3 and CD49b identifies T regulatory Tr1 cells (Gagliani et al., 2013), whereas LAG3 and CD49b are absent on Treg cells. Our confocal microscopy indicates that the IL-10 producing CD4 T cells have the phenotype of Tr1 cells. It has been shown earlier that IL-10 is also secreted by CD8^+^ T cells during *M.tb* infection (Cyktor et al., 2013; Moreira-Teixeira et al., 2017) and CD8 cells with a Tr1 have been described (Steinbrink et al., 1999; Gilliet and Liu, 2002). Thus, it is possible that there are also IL-10 producing CD8^+^ Tr1 cells in the lungs of old stressed mice. However, it is likely these CD8^+^ T cells are few in number, as we found only a few IL-10 producing cells that were not CD4^+^ T cells. Tr1 cells are antigen specific CD4^+^ T cells with regulatory activity that secrete large amounts of IL-10 and TGF-β and do not express FOXP3 (Roncarolo et al., 2006; Roncarolo et al., 2018). Differentiation of antigen-specific Tr1 cells has been shown to be dependent on antigen presentation by a subset of IL-10-producing dendritic cells (Wakkach et al., 2003; Gregori et al., 2010). In addition to IL-10, IL-27 promotes the differentiation of Tr1 cells (Pot et al., 2009; Pot et al., 2011). In our study, IL-27 mRNA levels in the lung were higher at 60 days post infection than at 30 days in both stressed and non-stressed mice. Thus, our data suggest that while IL-27 is present in the lungs at 60 days of infections, differences in IL-27 production are not responsible for differences in CD4 T cell and IL-10 expression between young and old stressed mice. While our study points to a role for IL-10 secreting T cells in the loss of control in old stressed mice, more definitive single cell RNAseq analysis of T cell populations in the lungs of old stressed mice will be needed to define the transcriptome of these IL-10 producing T cells and to confirm these T cells have the phenotype of Tr1 cells.

Another question that needs to be addressed is how SDR stress prior to infection alters T cell immunity at the later stage of infection. The effects of SDR on spleen weight, plasma IL-6, release of monocytes into the circulation, and anxiety behavior last at least 8 days after the last SDR cycle but are diminished by day 24 (Wohleb et al., 2014). This suggests effects of SDR activation of the SNS in the lung and draining lymph nodes will persist during early period in which the adaptive immune system is activated. The first 21 days after *M.tb* is the critical period for the differentiation of *M.tb* antigen specific CD4 T cells. The presence of IL-10 during the first 21 days of *M.tb* infection has a long-term effect on the control of the infection. This was shown by treatment of CBA/J mice during the first 21 days with anti-IL-10R antibody to block effects of IL-10 (Cyktor et al., 2013). This early treatment with anti-IL10R antibody resulted in increased numbers of CD4^+^ and CD8^+^ T cells in the draining lymph node and significantly increased IFN-γ mRNA in the lung at day 21. Thus, IL-10 has a significant impact on the differentiation of T cells during the early stage of infection. The anti-IL-10R antibody treatment was halted at day 21, and the long-term effect on *M.tb* infection was examined 100 day later. The early anti-IL-10R treatment significantly decreased *M.tb* burden in the lung and led to formation of mature granulomas with fibrotic capsule.

During the first days of an *M.tb* infection, IL-10 production occurs primarily in myeloid cells, including monocytes, macrophages, and dendritic cells (Moreira-Teixeira et al., 2017). We propose that SDR activation of the SNS in the draining lymph nodes of old mice triggers the production of IL-10 by antigen presenting cells, resulting in differentiation of T cells into IL-10 secreting Tr1 cells, which then migrate to the lung. In this regard, studies have shown that norepinephrine signaling through the β2-adrenergic receptor increases rapid IL-10 production by macrophages and dendritic cells (Agac et al., 2018). IL-10 secreting human Tr1 cells are also induced by a combination of glucocorticoids and β2-agonists (Peek et al., 2005). Our analysis of *M.tb* infection at 60 days post infection, suggests that IL-10 production by T cells and production of IL-27 by macrophages will further expand differentiation of Tr1 cells, which eventually leads to decreased ability of stressed old mice to control the infection. Exactly how aging impacts the stress response remains elusive. One possible explanation is that SDR induces greater activation of the SNS in old mice, resulting in the increased levels of norepinephrine being produced. Aging may also increase the number of APCs that induce differentiation of IL-10 secreting T cells. Our previous study (Lafuse et al., 2019) showed that old mice have higher numbers of a CD11c^+^ CD11b^+^ AM subpopulation that phagocytosed more *M.tb* than the major CD11c^+^ CD11b^+^ AM population. We found that CD11c^+^ CD11b^+^ AM population preferentially express CCR7, which is required for migration to lymph nodes (Forster et al., 1999). This population also expresses high levels of β2-adrenergic receptor mRNA. Thus, we posit that this AM population is involved in trafficking of *M.tb* to the draining lymph node and the higher numbers of this AM population in old mice will lead to increased numbers of IL-10 secreting T cells in old SDR mice compared to young SDR mice.

A limitation to this study is that it does not contain a human component examining effects of psychological stress during tuberculosis in the elderly. Social disruption stress is a chronic stressor that creates anxiety behavior similar to chronic stress in humans (Kinsey et al., 2007). Chronic psychological stress in humans contributes to chronic inflammation that occurs with aging, leading to disease susceptibility. In the elderly, age-related neuroendocrine dysregulation can exacerbate the inflammatory response to the stress (Heffner, 2011). Chronic stress in humans has been shown to be associated with altered T cell cytokine response with decreased production of IFN-γ and increased production of IL-10 (Rink et al., 1998; Glaser R. et al., 2001). Human type-1 regulatory T cells capable of producing high levels of IL-10 have been described (Levings and Roncarolo, 2005). Further, CD4^+^ CD25^hi^ T regulatory cells are present in the lungs and blood of TB patients (Ribeiro-Rodrigues et al., 2006). Also, an *in vitro* studies demonstrated that human type-1 regulatory T cells that secrete IL-10, suppresses the T cell responses to *Mycobacterium bovis* BCG (Truscott et al., 2010). Although we do not have direct evidence for chronic stress induced type-1 regulatory T cells during human tuberculosis, these studies are supportive of their involvement.

In conclusion, we demonstrate that psychological stress in old mice creates an immune-suppressive environment in the lung, which has long-term effects on ability of the immune system to control an *M.tb* infection. Although our studies indicate that psychological stress in old mice increases the loss of control of the infection by increasing differentiation of IL-10 secreting T cells, further studies are needed to elucidate the mechanisms by which psychological stress alters T cell differentiation. Our studies are applicable to the understanding immune-based mechanisms that contribute to a broad range of lung diseases, for which aging and psychological stress commonly contribute to outcomes.

## Data availability statement

The original contributions presented in the study are included in the article/**Supplementary Material**. Further inquiries can be directed to the corresponding authors.

## Ethics statement

The animal study was reviewed and approved by The Ohio State University Institutional Animal care and Use Committee.

## Authors contributions

WPL, QW, NK, NS, SS, OA, and MVSR preformed the experiments, analyzed data, and generated figures. Experiments were supervised by WPL and MVSR. WPL and MVSR wrote an initial manuscript draft that was critically edited by JT and approved by all authors. All authors contributed to the article and approved the submitted version.

## Funding

This work was supported by a diversity supplement to National Institutes of Health grants P01-AG051428 (to JT and WPL), AI146252, AI146690 and AG 073720 (to MVSR), and P30-CA016068.

## Conflict of interest

The authors declare that the research was conducted in the absence of any commercial or financial relationships that could be construed as a potential conflict of interest.

## Publisher’s note

All claims expressed in this article are solely those of the authors and do not necessarily represent those of their affiliated organizations, or those of the publisher, the editors and the reviewers. Any product that may be evaluated in this article, or claim that may be made by its manufacturer, is not guaranteed or endorsed by the publisher.

## Abbreviations

AMs, alveolar macrophages; *M.tb*, *Mycobacterium tuberculosis*; BAL, bronchoalveolar lavage; SDR, social disruption stress; qRT-PCR, quantitative RT-PCR; NE, norepinephrine; TR1 cells, regulatory type 1 cells; TLR2, Toll Receptor 2.
