# Supplementary material for: Psychological stress creates an immune suppressive environment in the lung that increases susceptibility of aged mice to Mycobacterium tuberculosis infection
Source: Front Cell Infect Microbiol. 2022 Sep 16;12:990402. doi: 10.3389/fcimb.2022.990402 (PMC9523253; doi:10.3389/fcimb.2022.990402)
Supplement: Supplementary file 1 [file DataSheet_1.docx]

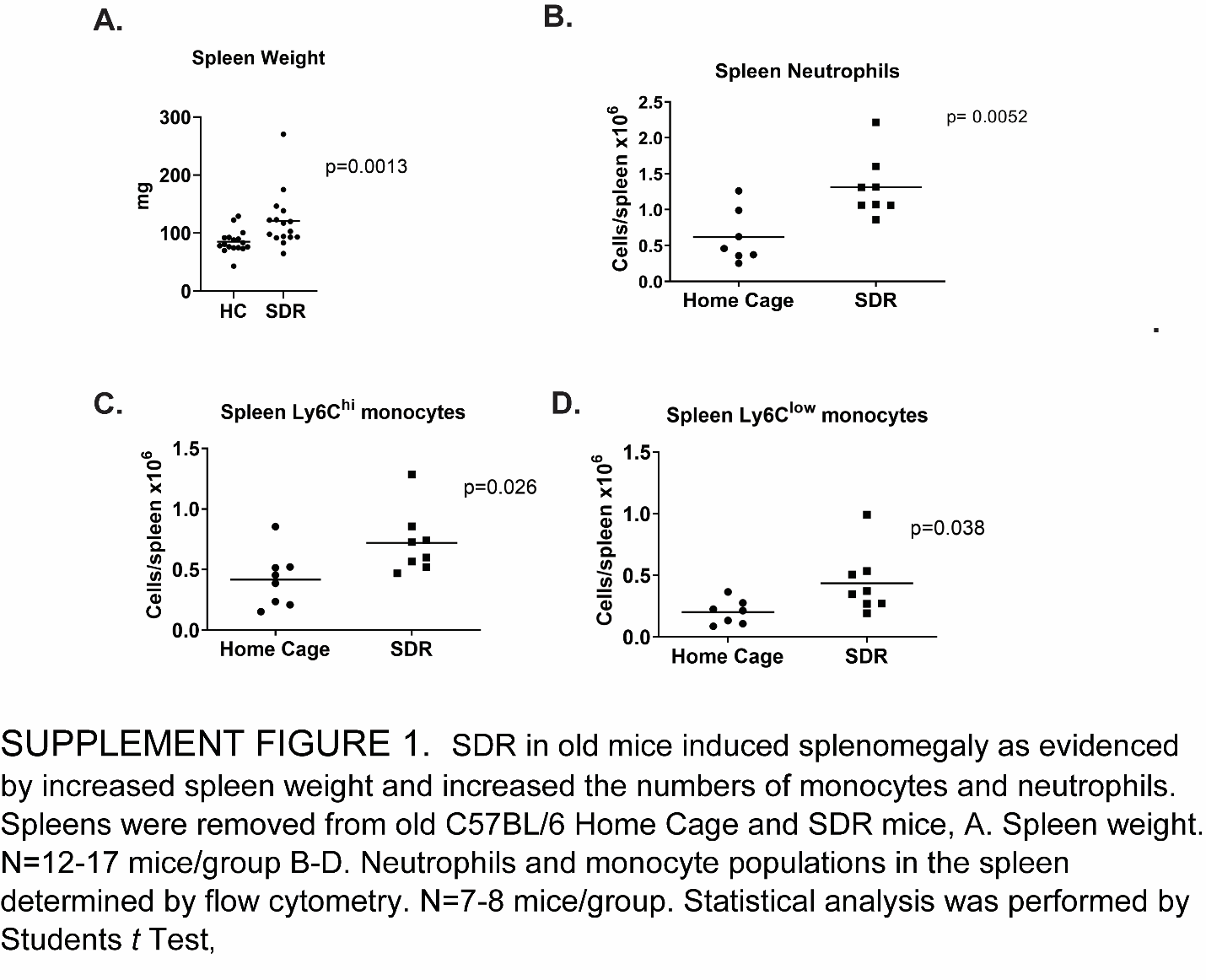


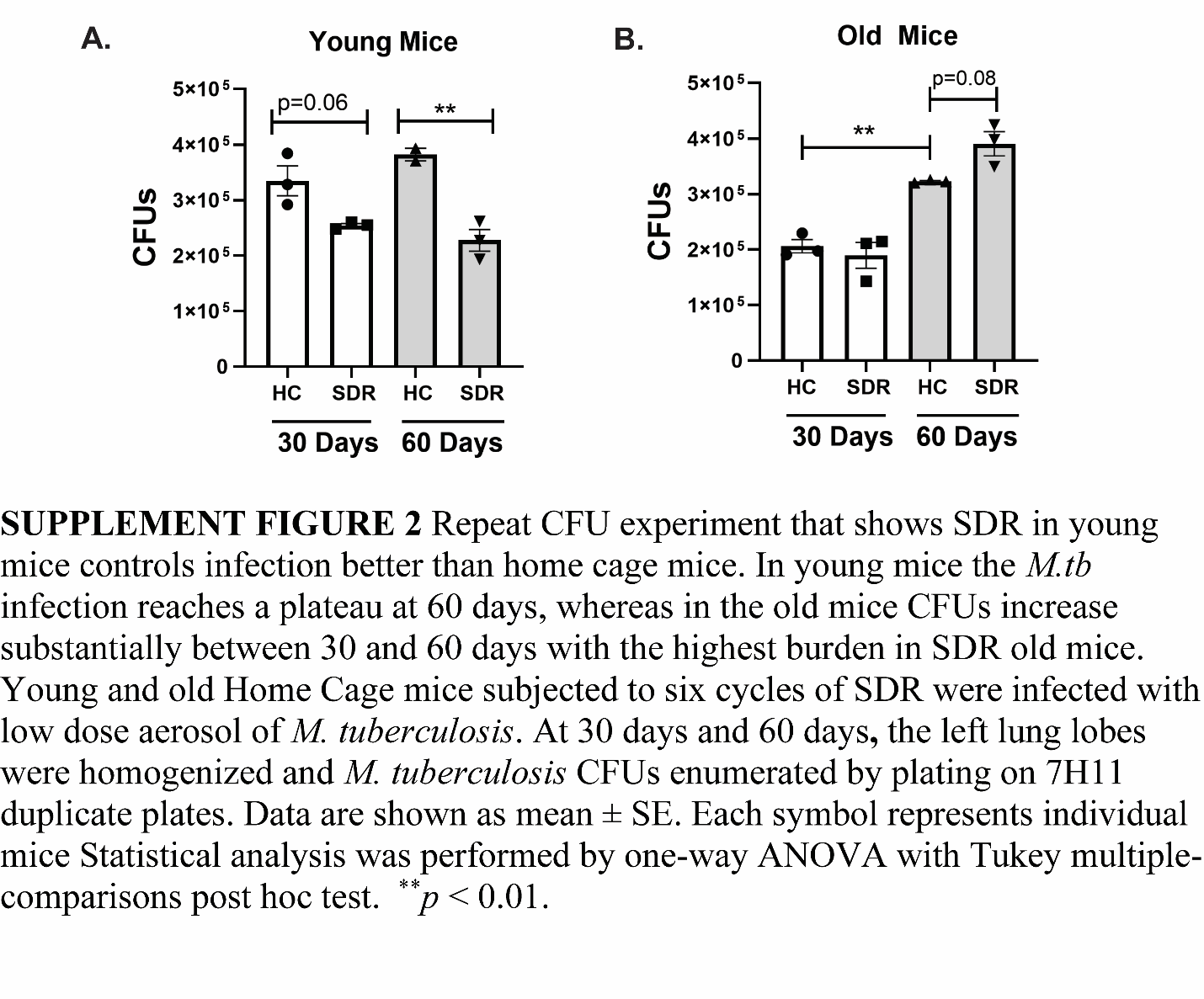


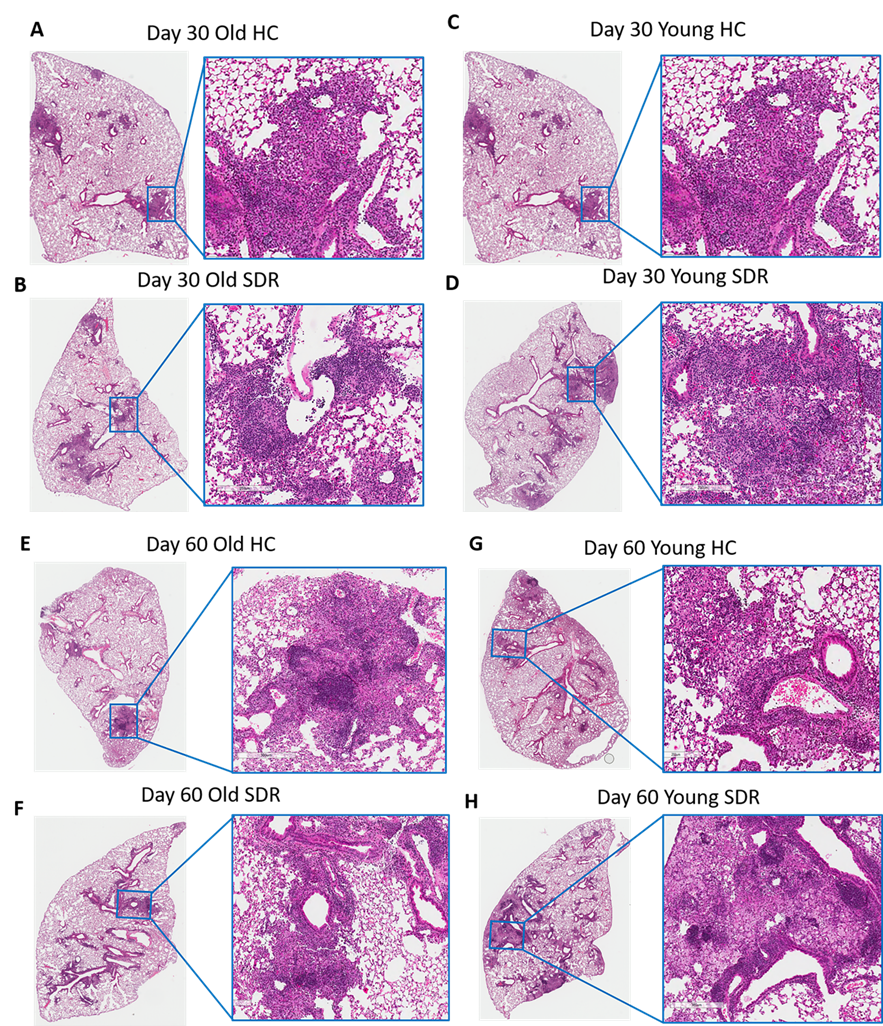


Supplemental Figure 3: Lung section from old home cage (A & E), SDR (B&F) and young home cage (C&G) and SDR (D&H) at day 30 and 60 respectively, were stained with H&E staining. Granulomatous regions were enlarged. Images shown are representative of 6 mice.

| **Gene** | **Forward** | **Reverse** |
| --- | --- | --- |
| beta-actin | TACAGCTTCACCACCACAGC | AAGGAAGGCTGGAAAAGAGC |
| CCL2 | TTAAAAACCTGGATCGGAACCAA | GCATTAGCTTCAGATTTACGGGT |
| CXCL2 | CCAACCACCAGGCTACAGG | GCGTCACACTCAAGCTCTG |
| IL-1β | TTCAGGCAGGCAGTATCACTC | CCACGGGAAAGACACAGGTAG |
| IL-6 | CACAAGTCCGGAGAGGAGAC | CAGAATTGCCATTGCACAAC |
| IL-10 | GCTCTTACTGACTGGCATGAG | CGCAGCTCTAGGAGCATGTG |
| IL12p35 | TGATGATGACCCTGTGCCTTGGTA | ATTCTGAAGTGCTGCGTTGATGGC |
| IL12p40 | TGGTTTGCCATCGTTTTGCTG | ACAGGTGAGGTTCACTGTTTCT |
| IL-17 | TTTAACTCCCTTGGCGCAAAA | CTTTCCCTCCGCATTGACAC |
| IL-21 | AATTCAATGCAGCACAGGCTAAGA | GTTCCCACCCACAGTGAACAATAA |
| IL-27p28 | CTGTTGCTGCTACCCTTGCTT | CACTCCTGGCAATCGAGATTC |
| TNFα | CATCTTCTCAAAATTCGAGTGACAA | TGGGAGTAGACAAGGTACAACCC |
| IFN-γ | ATGAACGCTACACACTGCATC | CCATCCTTTTGCCAGTTCCTC |
| Adgre1 | TGACTCACCTTGTGGTCCTAA | CTTCCCAGAATCCAGTCTTTCC |
| Jak3 | CCATCACGTTAGACTTTGCCA | GGCGGAGAATATAGGTGCCTG |
| Lcp2 | AGAGGACTTCCTGTCTGTATCAG | TGGACCCTCGATTCTTTCCATC |
| T-Bet | AGCAAGGACGGCGAATGTT | GGGTGGACATATAAGCGGTTC |
| GATA3 | CTCGGCCATTCGTACATGGA | GGATACCTCTGCACCGTAGC |
| RORγt | GACCCACACCTCACAAATTGA | AGTAGGCCACATTACACTGCT |
| TGF-β1 | CTCCCGTGGCTTCTAGTGC | GCCTTAGTTTGGACAGGATCTG |
| TGF-β3 | CAGGCCAGGGTAGTCAGAG | ATTTCCAGCCTAGATCCTGCC |

Supplemental Table 1: List of primers used for qRT-CPR in this studies.
